# Gender Dimorphism in Hepatic Carcinogenesis-Related Gene Expression Associated with Obesity as a Low-Grade Chronic Inflammatory Disease

**DOI:** 10.3390/ijms232315002

**Published:** 2022-11-30

**Authors:** Andrea G. Izquierdo, Marcos C. Carreira, Gemma Rodriguez-Carnero, Raquel Perez-Lois, Luisa M. Seoane, Felipe F. Casanueva, Ana B. Crujeiras

**Affiliations:** 1 Epigenomics in Endocrinology and Nutrition Group, Epigenomics Unit, Instituto de Investigacion Sanitaria de Santiago de Compostela (IDIS), Complejo Hospitalario Universitario de Santiago de Compostela (CHUS/SERGAS), 15706 Santiago de Compostela, Spain; 2 CIBER Fisiopatologia de la Obesidad y Nutricion (CIBERobn), 28029 Madrid, Spain; 3 Molecular Endocrinology Group, Instituto de Investigacion Sanitaria de Santiago de Compostela (IDIS), Complejo Hospitalario Universitario de Santiago de Compostela (CHUS/SERGAS), 15706 Santiago de Compostela, Spain; 4 Division of Endocrinology and Nutrition, Complejo Hospitalario Universitario de Santiago de Compostela (CHUS/SERGAS), 15706 Santiago de Compostela, Spain; 5 Endocrine Physiopathology Group, Instituto de Investigacion Sanitaria de Santiago de Compostela (IDIS), Complejo Hospitalario Universitario de Santiago de Compostela (CHUS/SERGAS), 15706 Santiago de Compostela, Spain

**Keywords:** adiposity, steatosis, inflammation, hepatic carcinogenesis, HCC, NASH, NAFLD, sex differences, hepatic sex-biased gene expression, circulating blood cells, biomarkers, survivin

## Abstract

Non-alcoholic fatty liver disease (NAFLD) and hepatocellular carcinoma (HCC) show clear evidence of sexual dimorphism, with a significantly higher incidence in males. Among the determining factors that could explain this sex-based difference, the specific distribution of fat by sex has been suggested as a primary candidate, since obesity is a relevant risk factor. In this context, obesity, considered a low-grade chronic inflammatory pathology and responsible for the promotion of liver disease, could lead to sexual dimorphism in the expression profile of genes related to tumor development. When we compared the expression levels of genes associated with the early stages of carcinogenesis in the liver between male and female diet-induced obesity (DIO) rats, we observed that the expression pattern was similar in obese male and female animals. Interestingly, the *SURVIVIN/BIRC5* oncogene showed a higher expression in male DIO rats than in female DIO and lean rats. This trend related to sexual dimorphism was observed in leukocytes from patients with obesity, although the difference was not statistically significant. In conclusion, this study evidenced a similar pattern in the expression of most carcinogenesis-related genes in the liver, except *SUVIVIN/BIRC5*, which could be a predictive biomarker of liver carcinogenesis predisposition in male patients with obesity.

## 1. Introduction

In mammals, sexual dimorphism appears in numerous biological processes as well as in parameters such as the incidence, prevalence and mortality of diseases [1,2]. The liver exhibits a high degree of sexual dimorphism under normal health conditions, with hundreds of genes that are differentially expressed between the two sexes [3,4], contributing to the physiology, homeostasis and metabolism of compounds [5,6,7]. These sex differences also interfere with liver function and the susceptibility, development and consequences of liver diseases, such as non-alcoholic fatty liver disease (NAFLD) or its most severe manifestation and the most common primary liver tumor, hepatocellular carcinoma (HCC) [8,9,10,11]. In fact, it has been described that in both NAFLD and HCC, there is clear evidence of sexual dimorphism in both rodents and humans, with a significantly higher incidence in males [8]. In almost all populations, regardless of etiologies, men have higher rates of liver cancer than women, with an estimated male:female ratio of 2 to 3:1 for HCC [12]; however, the reasons for this gender disparity are complex and not well understood. It is believed that it could probably be related to a set of behavioral and metabolic risk factors, environmental exposure, the biology of the tumor and the treatments received [13,14], as well as being due to the differences in sexual hormones in terms of the stimulating effect of androgens compared to the protective effect of estrogens in the development and progression of HCC [13,15,16].

In this regard, in recent years, several reports have suggested that sex-specific fat distribution could be one of the determining factors that could explain, at least in part, the different incidence of HCC between both sexes [17], as adiposity-related liver diseases increasingly emerge as the most common cause of chronic liver disease. In addition, it is known that the main cause of HCC is NAFLD associated with obesity [18,19,20,21], which is the most common form of chronic liver disease globally, affecting approximately 25–30% of the general population [22], 58% of individuals who are overweight and up to 90% of patients with obesity [20,23,24]. Thus, the prevalence of HCC is estimated to increase concomitantly with the current obesity epidemic, and is expected to account for approximately 90% of the >1 million cases of liver cancer estimated by 2025 [12,25,26].

Previous studies by our research group showed that the factors secreted by dysfunctional obese adipose tissue are responsible for triggering inflammation and oxidative stress, acting as potential promoters in the development of a favorable microenvironment for the initiation of carcinogenesis [27,28,29,30,31]. Specifically, when we analyzed the effect of excessive adiposity on the promotion of hepatocarcinogenesis in the liver of genetically obese rats, we observed that the regulation of genes related to tumor development depended directly on the state of adiposity and its effects on systemic inflammation and oxidative stress, even before the manifestation of a detectable tumor mass in the liver of these animals [27]. However, these results were observed in male animals, and the possibility that a gender bias interferes with the predisposition and future development of obesity-associated HCC is an open question that highlights the need for more studies in this field of research. To date, male subjects have been favored in human and animal biomedical research [32].

Considering the differences in the prevalence of liver disease between males and females, we hypothesized that the described effects of the excessive adiposity characteristic of obesity, such as inflammation and oxidative stress, in promoting hepatocarcinogenesis may be sexually dimorphic in the expression profile of the set of genes previously studied in relation to the early stages of tumor development. Therefore, the current study aimed to evaluate the differences due to sex in the expression levels of genes associated with the early steps of carcinogenesis in the liver of male and female rats with diet-induced obesity (DIO) and to explore whether this difference can be detected in blood leukocytes from patients with obesity.

## 2. Results

### 2.1. Sex-Biased Carcinogenesis-Related Gene Expression in Livers from Obese Rats

Statistically significant differences in body weight, fat mass, and fat-free mass were observed between the male and female rats (Table 1). As expected, in both male and female rats, the body composition analysis revealed that the obesity group had a higher body weight and fat mass than the lean group (Table 1). These parameters were significantly higher in male rats than in their female counterparts (Table 1).

**Figure 1:**
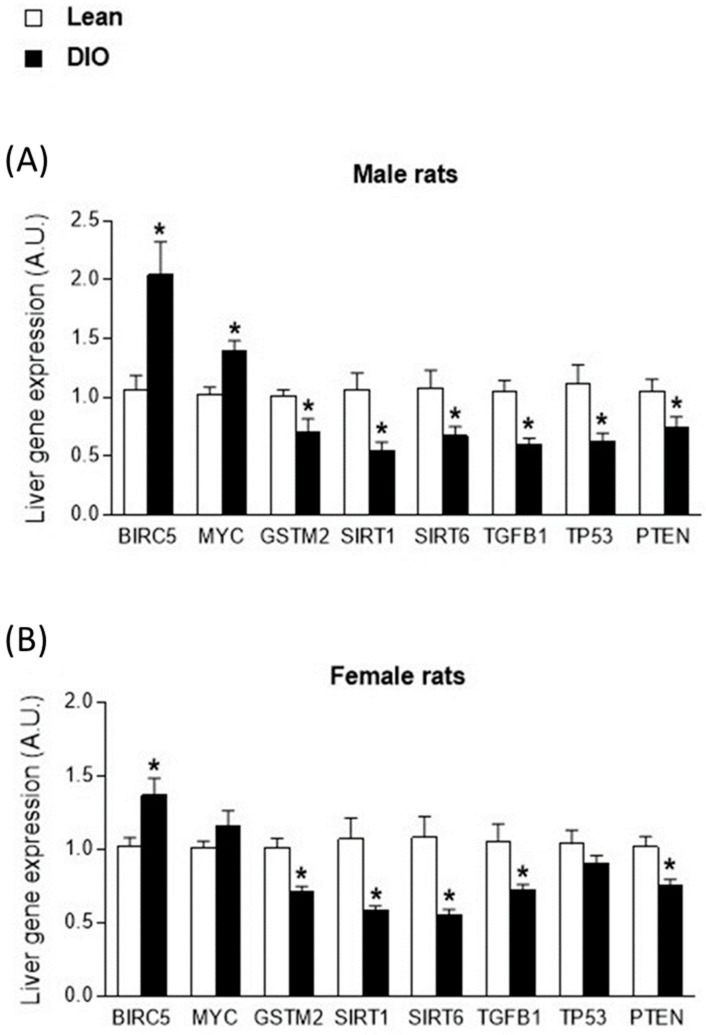
Hepatic expression of genes related to carcinogenesis in rats with diet-induced obesity (DIO) according to the obese phenotype and according to gender. (**A**) Gene expression levels in liver samples from male DIO rats. (**B**) Gene expression levels in liver samples from female DIO rats. Data are presented as mean ± standard error of the mean (SEM). * Statistically significant differences compared with control-lean group (*p* < 0.05). *Survivin/BIRC5*: survivin; *MYC*: MYC proto-oncogene; *GSTM2*: glutahione-S-transferase; *SIRT1*: sirtuin 1; *SIRT6*: sirtuin 6; *TGFB1*: transforming growth factor-beta 1; *TP53*: tumor protein p53; *PTEN*: phosphatase and tensin homologue.

**Figure 2:**
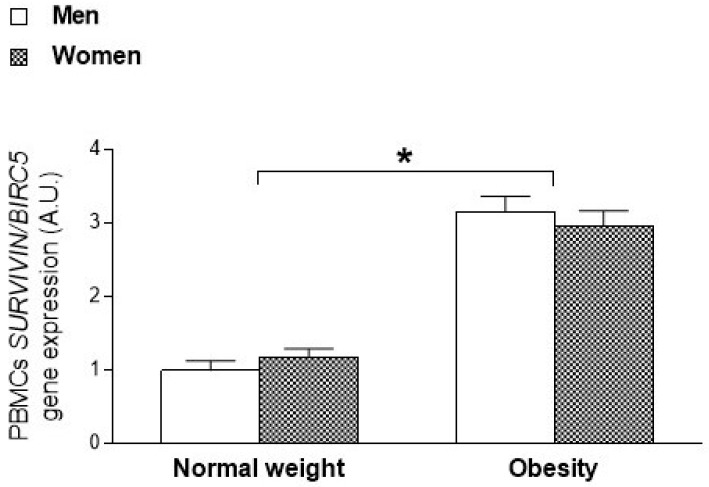
*Survivin/BIRC5* gene expression biased by sex in relation to the degree of adiposity. *Survivin/BIRC5* gene expression in PBMCs from patients with obesity compared to healthy individuals of normal weight according to gender. Data are presented as mean ± standard error of the mean (SEM). * Statistically significant differences compared with normal weight subjects (*p* < 0.05). *Survivin/BIRC5*: survivin; PBMCs: peripheral blood mononuclear cells.

Hepatic expression of the studied genes in relation to the onset of carcinogenesis was first evaluated by comparing rats with obesity and lean rats separated by sex (Figure 1A and Figure 2). In both male and female rats, the obesity group of animals showed an upregulation of the oncogenes *SURVIVIN/BIRC5* and *MYC*, together with a downregulation of genes linked to antioxidant protection, *GSTM2*, *SIRT1* and *SIRT6*, and the tumor suppressors *TGFB1*, *TP53* and *PTEN* (Figure 1A,B). Specifically, these differences were statistically significant for all the genes studied, except for the *MYC* and *TP53* genes analyzed in the livers of female rats (Figure 1B).

To further evaluate the effect of sex on gene expression, the interaction between the obese phenotype and sex was evaluated (Table 2). Under these conditions, independent of sex, statistically significant differences according to the obesity phenotype were found in all studied genes. Considering the sex of the animals, the analysis did not reveal a differential gene expression biased by sex, except in the *SURVIVIN/BIRC5* oncogene, which showed higher levels of gene expression in males than in females (*p* = 0.041) (Table 2).

### 2.2. Sexual Dimorphism in SURVIVIN/BIRC5 Oncogene Expression According to the Degree of Adiposity in Animals and Humans

Taking into account that male rats showed higher fat mass than female (Table 1), an ANCOVA was performed, adjusting for fat mass of the animals at the end of the study. In this analysis, the interaction between the obesity phenotype and sex for the expression of *SURVIVIN/BIRC5* was statistically significant (*p* = 0.026).

The *SURVIVIN/BIRC5* oncogene expression data were then segmented by the obesity and lean groups of rats, and an analysis was performed in relation to sex and adjusted for final fat mass. Interestingly, the expression of *SURVIVIN/BIRC5* was higher in male DIO rats than in female or lean animals, and these differences were statistically significant (*p* = 0.032).

Additionally, sexual dimorphism in *SURVIVIN/BIRC5* expression was evaluated in the blood leukocytes of patients with obesity and normal weight healthy individuals to assess the translation from animal results to humans. Transcriptional data for the *SURVIVIN/BIRC5* oncogene were obtained from a previous cohort in our research group (Table 3). Under these conditions, a previous analysis of *SURVIVIN/BIRC5* expression in PBMCs revealed significantly higher levels in patients with obesity (*p* < 0.001) [30], mirroring the results observed in the livers of rats (Figure 1). In the current study, we analyzed the effect of sex by an ANOVA performed in relation to the degree of adiposity and sex of the subjects. No statistically significant differences were observed in the levels of *SURVIVIN/BIRC5* between male and female patients (*p* = 0.951), but higher transcript levels of this gene were observed in subjects with obesity than in normal weight subjects of both sexes (Figure 2).

## 3. Discussion

The present study demonstrated in a DIO animal model that the expression pattern of a set of genes involved in the early phases of carcinogenesis in the liver based on obesity status was similar in male and female animals. In male and female rats with obesity, we found an overexpression of the oncogenes *SURVIVIN/BIRC5* and *MYC*, which are involved in cell proliferation and the inhibition of apoptosis [33,34]; a decrease in the transcriptional levels of the *GSTM2*, *SIRT1* and *SIRT6* genes, which are involved in protection against cellular oxidative stress and in the repair of DNA damage [35,36]; and a decrease in the tumor suppressors *TGFB1*, *TP53* and *PTEN*, which are involved in the regulation of cell growth [37,38,39]. Further analysis revealed that the *SURVIVIN/BIRC5* oncogene, which is overexpressed in most tumors [40,41], showed statistically higher transcriptional levels in male DIO rats than in female DIO rats and lean animals. These data suggest that gene expression of the *SURVIVIN/BIRC5* oncogene could be considered as a potential biomarker of increased susceptibility to liver disease and subsequent development of HCC in obese men.

Several epidemiological studies have shown that the excessive accumulation of body fat, characteristic of obesity and leading to low-grade systemic inflammation, is a relevant risk factor that contributes to the appearance of NAFLD and the subsequent development of HCC [42,43], and it has been suggested that the increase in its prevalence is concomitant with the growing epidemic of obesity [44,45,46]. Furthermore, extensive literature suggests that chronic liver disease is influenced by sexual dimorphism. Recent studies have revealed significant differences between men and women in the prevalence, risk factors, pathophysiology, complications and treatments of NAFLD [17,47,48]. HCC is the fifth most common malignancy in men and the eighth in women [47,48]. Compared to women, men have a higher incidence of NAFLD, exhibit greater accumulation of visceral fat, experience more severe liver fibrosis and have a higher incidence of liver cancer. Furthermore, once NAFLD occurs, women show more rapid progression of liver fibrosis and higher levels of liver cell damage and inflammation [17,49]. However, despite the evidence in the literature, the underlying molecular mechanisms of sexual dimorphism in NALFD are not clear. Therefore, evaluating whether these sex differences should be considered in the prevention and personalized treatment of NAFLD and HCC is a necessary approach in the near future. In this context, new advances in understanding sexual dimorphism in the liver provide exciting clues about sex differences in NAFLD pathogenesis, which could inspire new therapeutic strategies. Furthermore, as obesity contributes greatly to the overall burden of NAFLD and HCC [20,21,44], there is an urgent need to delineate the molecular mechanisms of sex-biased obesity-associated hepatocarcinogenesis risk. Consequently, the present study evaluated gender dimorphism in the expression of a set of genes related to the first steps of carcinogenesis in the liver of rodents according to the degree of adiposity. This work revealed that the differences detected in the expression levels of most of the genes studied related to obesity were independent of sex, suggesting that differential gene expression is modulated by the excess adiposity characteristic of obese individuals, as previously proposed [27,28].

Interestingly, among the genes studied, we detected a relevant gender dimorphism in the obesity-related expression of the *SURVIVIN/BIRC5* oncogene. Specifically, significantly higher transcriptional levels of *SURVIVIN/BIRC5* were detected in male rats with obesity than in female rats with obesity and lean rats, after adjusting for fat mass. These results reflected an upregulation of *SURVIVIN/BIRC5* associated with excess adiposity, which was especially manifested in males. When we transferred these results to blood leukocytes in humans, involving a minimally invasive procedure considered useful and effective for reflecting tissue-specific gene expression, we observed that the expression of the *SURVIVIN/BIRC5* oncogene showed statistically significant differences due to the state of obesity, without being subject to a sex effect. These data were consistent with a previously detected increase in *SURVIVIN/BIRC5* in the visceral adipose tissue of male animals with obesity and blood leukocytes in humans [30]. In fact, in recent years, this oncogene has generated great interest as a biomarker of prognostic importance and therapeutic relevance in many malignant neoplasms such as HCC [33,50,51,52]. Thus, *SURVIVIN/BIRC5* overexpression has been associated with the onset and progression of several types of cancer [40,50,53,54,55], and, regarding HCC, the expression of *SURVIVIN/BIRC5* has been reported in 41% of cases and its high expression has been associated with a poor prognosis in patients [40,56,57,58]. In addition, to the best of our knowledge, this is the first study to evidence sex differences in the expression of the *SURVIVIN/BIRC5* oncogene associated with the state of obesity, which was detected even before the manifestation of a tumor mass in the liver. Our data show that *SURVIVIN/BIRC5* expression was particularly higher in males with obesity; therefore, the *SURVIVIN/BIRC5* oncogene could be postulated as a potential biomarker of susceptibility to HCC in male patients with obesity.

One limitation of this study could be the difference in age and body composition between the male and female rats. Consequently, the statistical analyses were adjusted for body fat mass. Moreover, the lack of differences between sexes in the expression of *SURVIVIN/BIRC5* in blood leukocytes could be due to the relatively small sample size in the human cohort. Another limitation could be the lack of data about other cancer risk factors, such as tobacco [59,60] or saturated fats [59,61] consumption among study patients, which could be factors that influenced the results. However, the trend in gene expression was similar to that observed in the animal model, suggesting that smoking was not a relevant confounding factor in this analysis. Regarding saturated fat consumption, a previous study from our research group demonstrated that excess adiposity is the main factor involved in the susceptibility to carcinogenesis rather than a high-fat diet [28].

## 4. Methods and Materials

### 4.1. Animals with Diet-Induced Obesity (DIO)

Twenty male and twenty female Sprague-Dawley rats (6–8 weeks of age) were obtained from the central animal facility at the University of Santiago de Compostela. After an acclimatization period (1 week), the animals were randomly distributed into two groups: (a) the lean or control group (n = 10 males/females) was fed *ad libitum* with a standard diet (SAFE-Panlab, Barcelona, Spain) consisting of 5.5% lipids, 23% protein and 70% carbohydrates; (b) and the obese or DIO group (n = 10 males/females) was provided *ad libitum* access to a high-fat diet (HFD) (Open Source Diets, Research Diets; Brogaarden, Denmark, Reference D 12492) composed of 60% fat, 20% protein and 20% carbohydrates.

The rats were housed in air-conditioned rooms (22–24 °C) under a controlled light–dark cycle (12:12 h) with free access to food and water. Body weight and food intake were measured periodically throughout the experimental period (9 weeks). Body composition was assessed weekly in all animals by nuclear magnetic resonance (NMR) using an EchoMRI-700 system (Echo Medical Systems, Houston, TX, USA) (Table 1). After the 9-week experimental period, the animals were sacrificed by decapitation, their livers were excised, immediately frozen on dry ice and stored at −80 °C until RNA was extracted and analyzed.

### 4.2. Patients with Obesity and Normal Weight Individuals

The study included a group of healthy subjects with normal weight (n = 29; 10 men; 40.2 ± 8.9 years, 23.5 ± 2.3 kg/m^2^) and a group of patients with obesity (n = 22; 10 men, 42.5 ± 10.8 years, 38.04 ± 6.9 kg/m^2^) (Table 3). All participants were in good health according to their medical history, a physical examination and routine hematologic and biochemical laboratory test results. The participants reported no use of vitamin supplements, mineral supplements or regular prescription medications during the previous three months. Blood samples were obtained after overnight fasting and peripheral blood mononuclear cells (PBMCs) were isolated with differential centrifugation using a Polymorphprep (Axis Shield PoC AS, Norway). The cell pellet was resuspended in TRIzol reagent (Invitrogen, Carlsbad, CA, USA) and immediately frozen at −80 °C until RNA extraction and analysis.

### 4.3. Gene Expression Assessment

RNA was isolated using TRIzol (Invitrogen, Carlsbad, CA, USA) from liver samples of rats and from the blood leukocytes derived from subjects according to the manufacturer’s recommendations. RNA concentration was measured using a Nanodrop 2000 spectrophotometer (Thermo Scientific, Waltham, MA, USA). From the total extracted RNA, 1 µg was purified with a DNase treatment using a DNA-free kit as a template (Thermo Scientific, USA), and first-strand cDNA was synthesized using a High-Capacity cDNA Reverse Transcription Kit (Applied Biosystems, Waltham, MA, USA).

Gene expression was assessed using real-time quantitative polymerase chain reaction (RT-qPCR) and was performed using a TaqMan Universal PCR Master Mix, TaqMan Probes (Supplementary Table S1) and the Step OnePlus Real-Time PCR System (Applied Biosystems, USA). All experiments were performed in duplicate. For data analysis, gene expression levels were normalized to the levels of the housekeeping gene (beta-actin or GAPDH) and expressed as the average value for the control group calculated using the 2^−ΔΔCt^ relative quantitation method according to the manufacturer’s guidelines (Applied Biosystems, USA). RT-qPCR experiments were performed in compliance with the *Minimum Information for Publication of Quantitative Real-Time PCR Experiments* guidelines (http://www.rdml.org/miqe).

### 4.4. Statistical Analysis

Normal distributions were explored using the Kolmogorov–Smirnov test and Shapiro–Wilk test. Differences in body weight, body composition and gene expression levels among groups comprising subjects with normal weight and obesity were evaluated using a Student’s *t*-test. An analysis of variance (ANOVA) or analysis of covariance (ANCOVA), adjusted for sex or the amount of fat mass at the end of the study, was used to analyze the differences between groups of obese and lean rats.

Data are reported as the mean ± standard error of the mean (SEM) or means ± standard deviation (SD). All values were considered statistically significant when *p*-value < 0.05, and a *p*-value ≤ 0.1 was considered a trend for significance. Statistical analysis was performed using GraphPad Prism 7 (GraphPad Software Inc., San Diego, CA, USA) and SPSS 25 software (SPSS Inc., Chicago, IL, USA) for Windows 10 (Microsoft, Redmond, WA, USA).

## 5. Conclusions

The findings of the present study demonstrated the effect of obesity per se on the deregulation of the expression of genes involved in the early steps of hepatic carcinogenesis, even before the manifestation of a tumor mass in the liver; these findings were consistent with previous data from our research group on an animal model of genetic obesity using male Zucker rats [27]. Our results showed that the regulation of most of the genes studied in relation to the early process of liver carcinogenesis depended directly on the state of adiposity and did not show significant differences with respect to the sex of the subjects, with the exception of the *SURVIVIN/BIRC5* oncogene. To the best of our knowledge, this work has shown for the first time that the upregulation of *SURVIVIN/BIRC5* in obese subjects is significantly higher in males, which highlights the potential role of the *SURVIVIN/BIRC5* oncogene as a predictive biomarker of susceptibility to HCC in male patients with obesity.

## Figures and Tables

**Table 1:** Characteristics of animal experimental groups.

	Lean		Obese	
Sex	Male	Female	Male	Female
**n**	10	10	10	10
**Age (weeks)**	8	6	8	6
**Body weight (g)**	562.3 ± 15.2	226.4 ± 10.8 ^#^	663.5 ± 76.4 *	245.8 ± 10.3 *^,#^
**Fat mass (g)**	52.4 ± 15.6	16.0 ± 4.26 ^#^	146.8 ± 59.7 *	30.4 ± 5.32 *^,#^
**Fat mass (%)**	9.30 ± 2.67	7.02 ± 1.71 ^#^	21.6 ± 6.50 *	12.4 ± 2.12 *^,#^
**Fat-free mass (g)**	420.0 ± 16.1	173.3 ± 10.1 ^#^	424.7 ± 27.6	170.6 ± 8.64 ^#^
**Fat-free mass (%)**	74.7 ± 2.34	76.6 ± 4.08	64.5 ± 6.23 *	69.4 ± 2.73 *^,#^

Data shown are mean ± SD (standard deviation). **^#^** Statistically significant differences compared with male rats. * Statistically significant differences compared with lean group counterparts.

**Table 2:** Differences in hepatic expression levels of genes related to carcinogenesis in DIO rats according to group and sex.

	Lean		Obese		ANOVA *p*-Value		
GENE	Male	Female	Male	Female	Group	Sex	Group * Sex
***SURVIVIN/BIRC5***	1.06 ± 0.39	1.02 ± 0.19	2.04 ± 0.89	1.37 ± 0.37	<0.001 *	0.041 *	0.068
***MYC***	1.02 ± 0.20	1.01 ± 0.15	1.40 ± 0.24	1.16 ± 0.33	0.003 *	0.144	0.183
***GSTM2***	1.01 ± 0.16	1.02 ± 0.18	0.70 ± 0.34	0.71 ± 0.11	<0.001 *	0.931	0.981
***SIRT1***	1.06 ± 0.46	1.07 ± 0.44	0.51 ± 0.24	0.59 ± 0.09	<0.001 *	0.807	0.899
***SIRT6***	1.08 ± 0.46	1.08 ± 0.45	0.67 ± 0.25	0.55 ± 0.12	<0.001 *	0.604	0.604
***TGFB1***	1.05 ± 0.30	1.06 ± 0.38	0.60 ± 0.17	0.72 ± 0.12	<0.001 *	0.424	0.503
***TP53***	1.11 ± 0.50	1.04 ± 0.30	0.62 ± 0.23	0.91 ± 0.16	0.004 *	0.323	0.085
***PTEN***	1.05 ± 0.33	1.02 ± 0.22	0.74 ± 0.30	0.75 ± 0.14	0.001 *	0.911	0.797

Data shown are mean ± SD (standard deviation). *Survivin/BIRC5*: survivin; *MYC*: MYC proto-oncogene; *GSTM2*: glutahione-S-transferase; *SIRT1*: sirtuin 1; *SIRT6*: sirtuin 6; *TGFB1*: transforming growth factor-beta 1; *TP53*: tumor protein p53; *PTEN*: phosphatase and tensin homologue. * Statistically significant differences (*p* < 0.05).

**Table 3:** Anthropometric parameters of study subjects.

	Normal Weight		Obesity	
	Men	Women	Men	Women
**n**	10	19	10	12
**Age (years)**	40.2 ± 8.82	33.5 ± 9.00	42.50 ± 10.8	35.7 ± 10.8
**Height (m)**	1.73 ± 0.06	1.65 ± 0.07	1.74 ± 0.05	1.64 ± 0.06
**Body weight (kg)**	67.2 ± 7.81	57.6 ± 4.07	114.2 ± 20.2 *	113.2 ± 21.6 *
**BMI (kg/m^2^)**	23.5 ± 2.33	21.4 ± 1.72	38.0 ± 6.90 *	41.9 ± 7.70 *
**WC (cm)**	86.2 ± 7.66	76.9 ± 7.89	120.8 ± 19.6 *	122.8 ± 15.5 *

Data shown are mean ± SD (standard deviation). Abbreviations: BMI, Body Mass Index; WC, waist circumference. * Statistically significant differences compared with normal weight group (*p* < 0.05).

## Data Availability

The datasets generated for this study are available on request to the corresponding author.

## Supplementary Materials

The following supporting information can be downloaded at https://www.mdpi.com/article/10.3390/ijms232315002/s1.
