# Supplementary material for: Gender Dimorphism in Hepatic Carcinogenesis-Related Gene Expression Associated with Obesity as a Low-Grade Chronic Inflammatory Disease
Source: Int J Mol Sci. 2022 Nov 30;23(23):15002. doi: 10.3390/ijms232315002 (PMC9739425; doi:10.3390/ijms232315002)
Supplement: Supplementary file 1 [file ijms-23-15002-s001.zip › ijms-2000605-supplementary.pdf]

Supplementary Table S1. Probes for RTqPCR (TaqMan®) analysis.

GENE	REFERENCE	TARGET SP
SURVIVIN/ BIRC5	Rn00574012_m1,	Rat
	Hs04194392_s1	Human
MYC	Rn00561507_m1,	Rat
GSTM2	Rn00598597_m1,	Rat
SIRT1	Rn01428093_m1,	Rat
SIRT6	Rn01408249_m1,	Rat
TGFB1	Rn00572010_m1,	Rat
TP53	Rn00755717_m1,	Rat
PTEN	Rn00477208_m1,	Rat
ACTB	Rn00667869_m1,	Rat
GAPDH	Hs02758991_g1	Human
